# Larvicidal and antibacterial activity of aqueous leaf extract of Peepal (*Ficus religiosa*) synthesized nanoparticles

**DOI:** 10.1016/j.parepi.2020.e00166

**Published:** 2020-07-20

**Authors:** Namita Soni, Ramesh C. Dhiman

**Affiliations:** Environmental Epidemiology Division, ICMR-National Institute of Malaria Research, New Delhi, India 110077

**Keywords:** *Ficus religiosa*, ZnO NPs, TiO_2_ NPs, Larvicides, *Anopheles stephensi*, Antimicrobial

## Abstract

In this study, the zinc oxide nanoparticles (ZnO NPs) and titanium dioxide nanoparticles (TiO_2_ NPs) were synthesized using the aqueous leaf extract of *Ficus religiosa* (Peepal tree). The synthesized nanoparticles were tested as larvicides against the larvae of *Anopheles stephensi.* Further, the synthesized nanoparticles were tested as antibacterial agents against the *Escherichia coli* (gram negative) and *Staphylococcus aureus* (gram positive) bacteria. The synthesized nanoparticles were characterized with UV-visible spectroscopy, X-rays powder diffraction (XRD), transmission electron microscopy (TEM), scanning electron microscopy with energy dispersive X-ray spectroscopy (SEM-EDX). The larvicidal mortality was observed after 24 h and 48 h by probit analysis. The antibacterial activity was evaluated using the well diffusion method. The synthesized nanoparticles were irregular shape and varied size. The larvae of *An. stephensi* were found highly susceptible against the ZnO NPs than the TiO_2_ NPs and aqueous leaves extract. The highest mortality was observed in synthesized ZnO NPs against first to third instars of (LC_50_ 50, 75, and 5 ppm) and 100% mortality in fourth instars of *An. stephensi*. The higher zone of inhibition was occurred against the *E. coli*. This report of present investigation revealed that the rapid biological synthesis of ZnO NPs and TiO_2_ NPs using aqueous leaf extract of *F. religiosa* would be effective potential larvicides for mosquito control as well as antimicrobial agents with eco-friendly approach

## 1 Introduction

*F. religiosa* is commonly known as Peepal tree belonging to the family moraceae. It is also known as bodhi tree, pippala tree, peepul tree, pepal tree and ashwattha tree. It is a medicinal plant which is emerged as a good source of traditional medicine for the treatment of asthma, diabetes, diarrhea, epilepsy, gastric problems, inflammatory disorders, infectious and sexual disorders (Singh et al., 2011a). The leaves of *F. religiosa* contain tannic acid, leucine, isoleucine, methionine, tryptophan, threonine, glycine asparatic acid, serine and arginine, bark comprises of bergaptol and bergapten, the fruits consists of tyrosine and asparagines and seeds contain threonine, alanine and valine (Gupta and Singh, 2012), which are the good source of secondary metabolites used as larvicides and antimicrobial agents.

The larvicidal activity of crude hexane, ethyl acetate, petroleum ether, acetone, and methanol extracts of the leaf and bark of *Ficus racemosa* (Moraceae) has been assayed for their toxicity against the early fourth-instar larvae of *Culex quinquefasciatus* (Abdul Rahuman et al., 2008). The larvicidal efficacy of different extracts of *F. benghalensis* against *Cx*. *quinquefasciatus*, *Ae. aegypti* and *An. stephensi* has been investigated (Govindarajan, 2010). Further, larvicidal efficacy of different solvent leaf extracts of *F. benghalensis* against *Cx*. *tritaeniorhynchus* and *An. subpictus* has been determined (Govindarajan et al., 2011). Thereafter, the Larvicidal activity of Indian medicinal plants, *Commiphora berryi*, *Commiphora pentandra*, *Pelargonium graveolens*, *Thevetia peruviana*, *Sesamum indicum*, *Ficus microcarpa*, *Melia dubia*, *C. bonplandianus*, *F. religiosa* and *Croton lacryma* has been tested against *Ae. aegypti* mosquito (Deepa et al., 2015). The antimicrobial properties of *Ficus* extract have been reported (Mandal et al., 2000; Ao et al., 2008; Oyeleke et al., 2008; Annan and Houghton, 2008; Kuete et al., 2008; Kuete et al., 2009; Usman et al., 2009; Alimuddin et al., 2010; Lazreg Aref et al., 2010). Unfortunately, the secondary metabolites of plants have the slow reaction against the mosquitoes. Therefore, it is needed to develop the eco-friendly and rapid technology for the mosquito control as well as antimicrobial agent, so that people can be protected from the bacterial and vector borne diseases.

Recently, the use of metal nanoparticles is a great attention in this regards. The plants synthesized ZnO NPs and TiO_2_ NPs are the good, rapid and eco-friendly sources for mosquito control and antibacterial agents also. The synthesis and characterization of ZnO NPs (Singh et al., 2011b; Awwad et al., 2014; Shekhawat et al., 2014; Gnanasangeetha and Thambavani, 2014; Raju Kooluru and Sharada, 2014; Suganya et al., 2015; Noorjahan et al., 2015; Varghese and George, 2015; Manokari and Shekhawat, 2015; Manokari et al., 2016a, Manokari et al., 2016b; Fatimah et al., 2016; Sharmila Devi and Dhinesh, 2016; Pinto and Nazareth, 2016) and TiO2 NPs (Sundrarajan and Gowri, 2011; Rajakumar et al., 2012; Ganapathi Rao et al., 2015; Khadar et al., 2015; Valli and Geetha, 2015; Chatterjee et al., 2016; Mythreyi et al., 2016; Dobrucka, 2017; Patidar and Jain, 2017) using the plant extract has been reported.

Acaricidal, pediculocidal and larvicidal activity of ZnO NPs using wet chemical method has been reported (Kirthi et al., 2011). The antimicrobial activity of synthesized ZnO NPs using plants has been reported (Jeeva Lakshmi et al., 2012; Aswathi Sreenivasan et al., 2012; Divya et al., 2013; Mishra and Sharma, 2015; Raj et al., 2015; Narendhran and Sivaraj, 2016; Salih and Smail, 2016; Jeba Jane Ratney and Begila David, 2016; Paul et al., 2016).

The larvicidal activity of plant synthesized TiO_2_ NPs has been assessed (Rajakumar et al., 2015; Suman et al., 2015; Gandhi et al., 2016). The antimicrobial activity of synthesized TiO_2_ NPs using plants has been studied (Maurya et al., 2012; Malarkodi et al., 2013; Santhoshkumar et al., 2014; Murphin Kumar et al., 2014; Hariharan et al., 2017). Till now no review is available on the larvicidal and antibacterial activities of ZnO NPs and TiO_2_ NPs synthesized using aqueous leaf extract of Peepal (*F. religiosa*). In the present investigation, the ZnO NPs and TiO_2_ NPs were synthesized using the aqueous leaf extract of *F. religiosa* and access their larvicidal and antibacterial properties. This ZnO NPs and TiO_2_ NPs technology could be a rapid, green and, eco-friendly approach for mosquito control and used as antimicrobial also.

## 2 Materials and methods

### 2.1 Collection and leaf extract preparation

The fresh and green leaves of *F. religiosa* were collected from the nearest area of ICMR-National Institute of Malaria Research, India. The leaves were rinsed with tap water and then with distilled water to remove dust and other particles. The rinsed leaves were then air dried for 1–2 h. After then, approximately 20 g of leaves were cut into fine pieces and put into a 250 ml conical flask which containing 100 ml of distilled water. Boil the flask for 1 h at 50 °C on a magnetic stirrer. After 1 h, cooled the extract and filtered through the whatman-1 filter paper and store the leaves filtrate for the experiment.

### 2.2 Synthesis of ZnO and TiO_2_ NPs

The ZnO NPs were biosynthesized by co-precipitation method described by Singh et al. (2011a) with some modification. 20 ml of leaf extract was heated at 60 °C for 10 min on a magnetic stirrer. After then, 50 ml of 0.1 M of Zinc nitrate solution and 50 ml of 0.2 M sodium hydroxide solution were added drop wise under stirring. The mixture was continued stirred for 1 h on magnetic stirrer which resulting cream colored precipitate of zinc hydroxide formed. Then, the precipitate was collected by centrifugation at 4000 rpm for 15 min and washed with deionized water and ethanol. The ZnO NPs were collected after dried in hot air oven for 48 h at 45 °C.

The TiO_2_ NPs were biosynthesized by the following method described by Suman et al. (2015) with some modification. The aqueous solution of TiO(OH)_2_ (5 mmol/L) was prepared and used for synthesis of TiO_2_ NPs. 20 ml leaf extract of *F. religiosa* was boiled at 50 °C. Then 80 ml of aqueous solution of 5 mmol/L TiO(OH)_2_ were added in the leaf extract and boil for 4 h with continuous stirring. After then reduction mixture was centrifuged at 4000 rpm for 15 min and resulting pellet was collected. The TiO_2_ NPs were dried in hot air oven for 48 h at 45 °C.

### 2.3 Characterization of ZnO and TiO_2_ NPs

Optical properties of synthesized ZnO and TiO_2_ NPs were confirmed by UV–visible double beam spectroscopy (HALO DB-20) in 300–500 nm wavelength range. The XRD pattern of synthesized ZnO and TiO_2_ NPs were carried out using X-ray diffractometer (Bruker X-ray diffractometer D-8 Advance) Cu-Kα radiations (λ = 0.15406 nm) in 2θ range from 20° to 80°. The average size and shape of synthesized ZnO and TiO_2_ NPs were obtained by transmission electron microscopy (Tecnai G^2^). The morphology of synthesized ZnO and TiO_2_ NPs were examined by scanning electron microscope (model no. Zeiss EVO MA 10). The synthesized ZnO and TiO_2_ NPs were analyzed for elemental analysis by energy dispersive X-ray spectroscopy (Oxford Inca Energy 250).

### 2.4 Rearing of larvae

The larvae of *An. stephensi* were reared in deionized water containing glucose and yeast powder. The colony of *An. stephensi* was maintained in the laboratory at 27 °C with relative humidity of (75 ± 5%) and 14 h of photoperiod using the standard method with some modifications (Geberg et al., 1994).

### 2.5 Bioassay, data management and statistical analysis

ZnO NPs and TiO_2_ NPs, synthesized using aqueous leaves extract of *F. religiosa* were tested for their killing activities against the *An. stephensi* larvae (I-IV instar). The bioassay was assessed using the standard method (World Health Organization, 2005). *An. stephensi* larvae were placed in a container in micro-free deionized water. After that, ZnO and TiO_2_ NPs with different test concentrations in 100 mL deionized water were prepared in 250 mL beakers. Bioassays were conducted separately at five different concentrations using serial dilution method, of synthesized ZnO and TiO_2_ NPs (25, 50, 100, 150 and 250 ppm). To test the larvicidal activity of ZnO and TiO_2_ NPs, 20 larvae were separately exposed to 100 mL of test concentration. Similarly, the control (without ZnO and TiO_2_ NPs) was run to test the natural mortality. The experiments were replicated thrice to validate the results. Thereafter, we examined their mortality after 24 h and 48 h. The data on the efficacy was subjected to probit analysis (Finney, 1971). The control mortality was corrected by Abbott's formula (Abbott, 1925).

### 2.6 Antibacterial activity of ZnO and TiO_2_ NPs

The antibacterial activity of synthesized ZnO and TiO_2_ NPs was evaluated against *E. coli* and *S. aureus*. The antibacterial activity was determined using the well diffusion method. The wells were prepared on plates with Muller-Hinton agar (MHA) medium. Then, the plates were seeded with different bacterial strains using sterile swab. Four wells were prepared using gel puncture in each plate. Each well was loaded with 50 μL of different concentration of ZnO and TiO_2_ NPs (50, 150, 250 and 500 ppm). Then, the plates were incubated at 35 °C for 24 h and zone of inhibition was observed.

## 3 Results and discussion

### 3.1 Proposed mechanism of synthesis of ZnO and TiO_2_NPs through *F. religiosa*

Based on the experimental work that has been done, there are series of chemical reaction that takes place. The complete hydrolysis of zinc nitrate and dihydroxy(*oxo*)titanium with the aid of *F. religiosa* aqueous leaves extract solution should result in the formation of ZnO and TiO_2_ nanoparticles. The richly available carbohydrates, tannin, alkaloids, steroids, terpenoids, saponin, reducing sugar and favonoids in the plant extract acted as stabilizing and capping agents, respectively. Hence, the proposed principle of formation of ZnO and TiO_2_ NPs involves the ionization of zinc nitrate and hydroxylation of dihydroxy(*oxo*)titanium in an aqueous medium to give Zn^2+^ which was reduced by phytochemical principle present in the aqueous extract of *F. religiosa*, to generate ZnO, which further aggregates to ZnO and TiO_2_ NPs as shown in Eq. 1 and Eq. 2.

**Figure:**
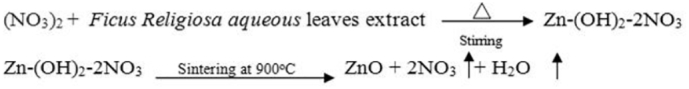

TiO(OH)_2_ + *Ficus religiosa* leaves extract$\frac{∆}{\;\mathrm{Stirring}}$ TiO_2_ + H_2_O↑.

### 3.2 UV-visible analysis

**Fig. 1:**
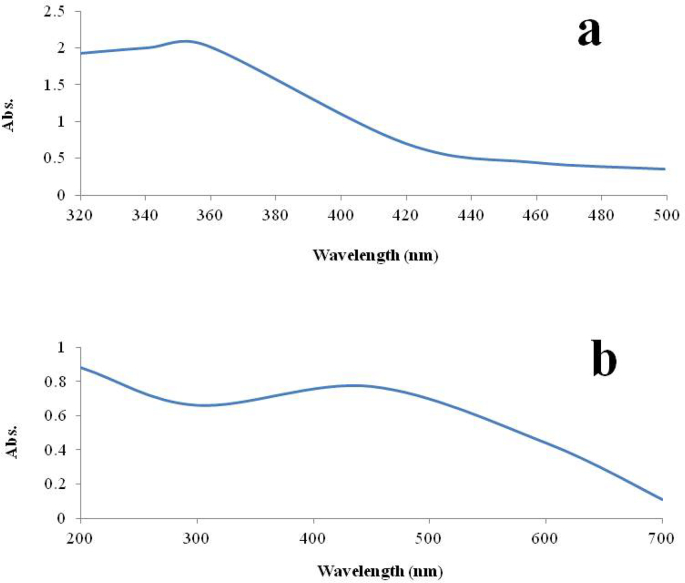
UV-visible spectrum of aqueous leaves extract synthesized (a) ZnO NPs and (b) TiO_2_ NPs.

The formation of ZnO and TiO_2_ NPs during the synthesis can be observed visually. Fig. 1a is the UV–vis absorption spectrum of ZnO NPs dispersed in deionized water and the figure shows the absorption peak at 358 nm. Shah et al. (2015) stated that the UV absorption spectrum for synthesized ZnO NPs was recorded at 330 nm. Similar results were observed by Yedurkar et al. (2016). Fig. 1b shows the UV-vis absorption spectrum of TiO_2_ NPs with an absorption peak at 450 nm. Valli and Geetha (2015) observed the UV absorption spectrum for synthesized TiO_2_ NPs at 447.3 nm.

### 3.3 XRD analysis

**Fig. 2:**
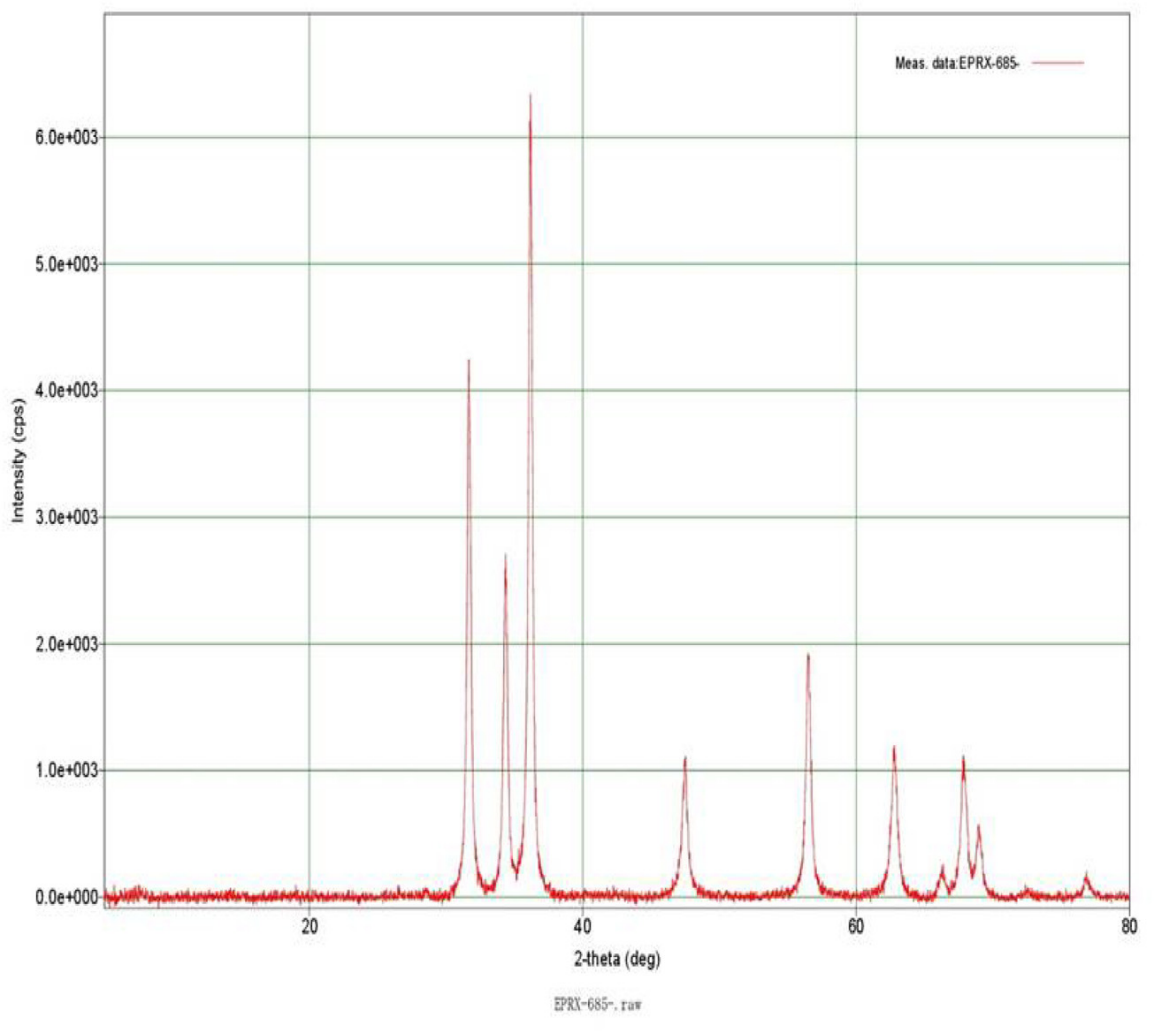
XRD pattern of synthesized ZnO NPs.

The structure of ZnO and TiO_2_ NPs were determined in this study using a powder diffraction system with Cu-Ka x-ray tube (λ = 1.541836 A) was used. Fig. 2 depicts the XRD pattern of synthesized ZnO NPs scanned at 2θ range from 0 to 80 degree. Diffraction peaks at 31.66°, 34.34°, 36.15°, 47.45°, 56.46°, 62.72°, 67.86°, 68.97°, 76.79° can be assigned to (110), (002), (101), (102), (110), (103), (112), (201) and (202) plane. The strong and narrow peak denotes that the product has well crystalline nature of particles. Narendhran and Sivakumar, (2016) recorded the X-ray diffraction of ZnO NPs synthesized using the L. *aculeate*. The peaks at 2θ values of 31.79°, 34.42°, 36.26°, 47.59°, 62.80°, 65.84°, 67.96°, 68.30°, 72.12° and 76.53° correspondence to the crystal planes of (100), (002), (101), (102), (110), (103), (200), (112), (201), (004) and (202) of ZnO NPs. Similar results were reported by Vanathi et al. (2014) in which particles were synthesized using *E. crassipes* leaf extract.

**Fig. 3:**
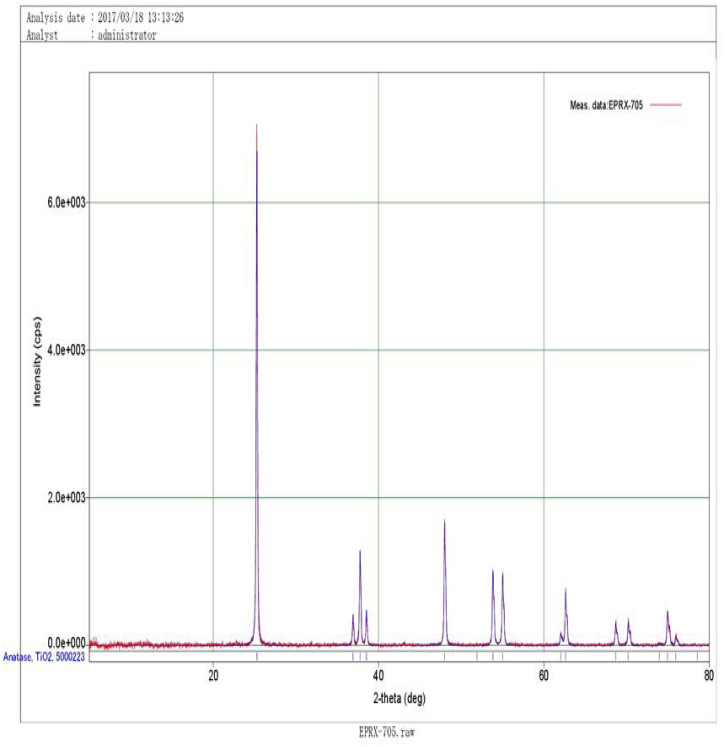
XRD pattern of synthesized TiO_2_ NPs.

Fig. 3 shows the XRD pattern of synthesized TiO_2_ NPs scanned at 2θ range from 0 to 80 degree. Diffraction peaks at 25.28°, 36.91°, 53.85°, 55.03°, 62.6°, 68.70° and 75.1° can be assigned to (110), (101), (211), (220), (204), (112) and (215) plane. Similar results were reported by Khadar et al. (2015).

### 3.4 TEM analysis

**Fig. 4:**
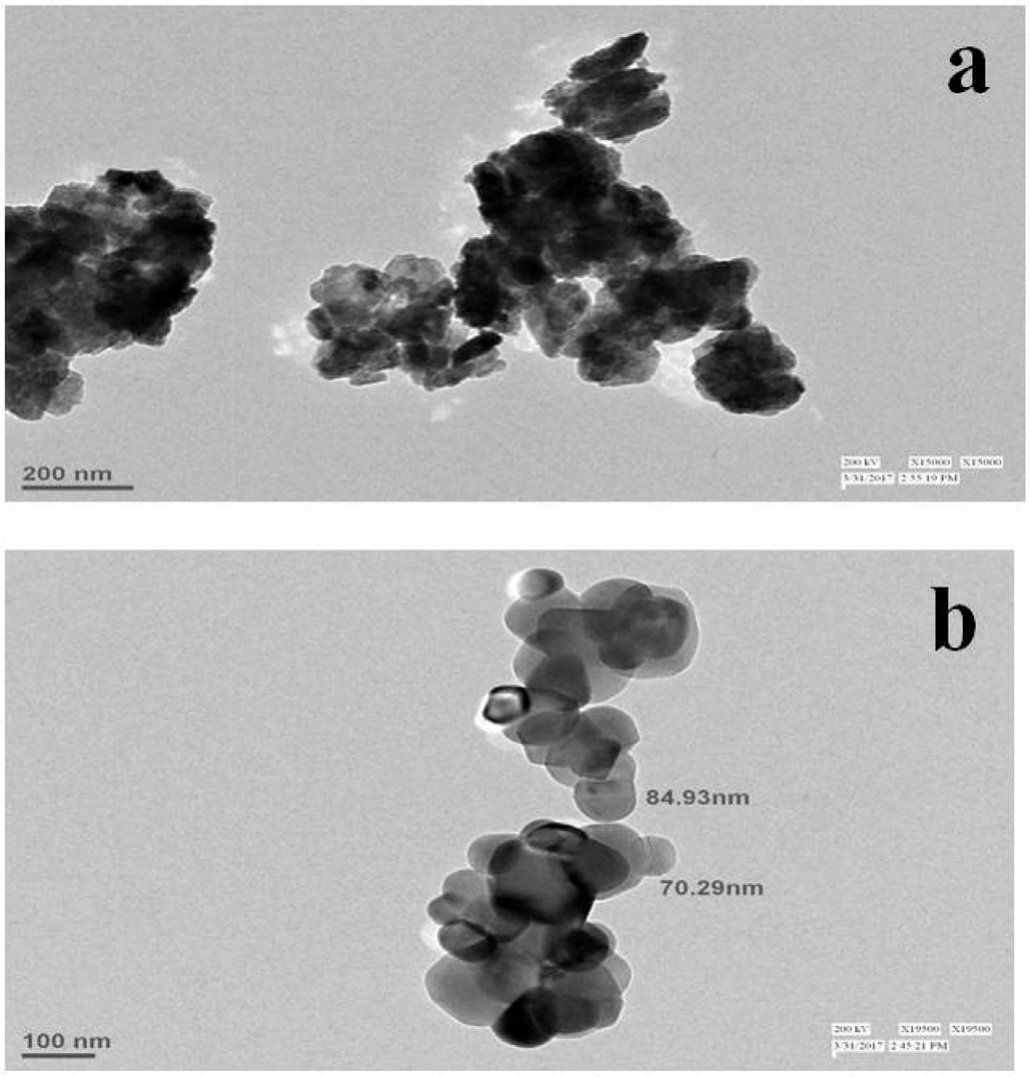
TEM images of synthesized (a) ZnO NPs and (b) TiO_2_ NPs.

The shape and size of synthesized ZnO and TiO_2_ NPs were obtained using the TEM. Fig. 4a shows the TEM images of synthesized ZnO NPs, which depict the irregular shape and varied size nanoparticles. Fig. 4b depicts the TEM images of TiO_2_ NPs, which were spherical in shape and size from 70.29 to 84.93 nm with calculated size of 77.61 nm and polydisperse. Zahir et al. (2014) recorded the TEM images of the synthesized Ag NPs and TiO_2_ NPs spherical, quite polydisperse and individual particles showed an average size of 12.82 ± 2.50 and 83.22 ± 1.50 nm, respectively. Similar results were obtained by (Rajakumar et al., 2012) and previous work (Table 1).

**Table 1:** Size Comparison of ZnO and TiO_2_ nanoparticles synthesized using the plants.

Plant species	Plant part used	Synthesized NPs	Size (nm)	References
*Azadirachta indica, Emblica Officinalis*	leaf	ZnO	51, 16	Gnanasangeetha and Thambavani, 2014
*Pyrus pyrifolia*	leaf	ZnO	45	Parthiban and Sundaramurthy, 2015
*Ixora coccinea*	leaf	ZnO	145.1	Yedurkar et al., 2016
*Passiflora caerulea*	Leaf	ZnO	200	Santhoshkumar et al., 2017
*Bauhinia tomentosa*	Leaf	ZnO	22–94	Sharmila et al., 2018
*Pandanus odorifer*	leaf	ZnO	90	Hussain et al., 2019
*Allium sativum*	Skin	ZnO	7.77	Modi and Fulekar, 2020
*Psidium guajava*	Leaf	TiO_2_	32.58	Santhoshkumar et al., 2014
*Cynodon dactylon*	Leaf	TiO_2_	13–34	Hariharan et al., 2017
*Glycosmis cochinchinensis*	Leaf	TiO_2_	45	Rosi and Kalyanasundaram, 2018
*Cassia fistula*	Leaf	TiO_2_	200	Swathi et al., 2019

### 3.5 SEM-EDX analysis

**Fig. 5:**
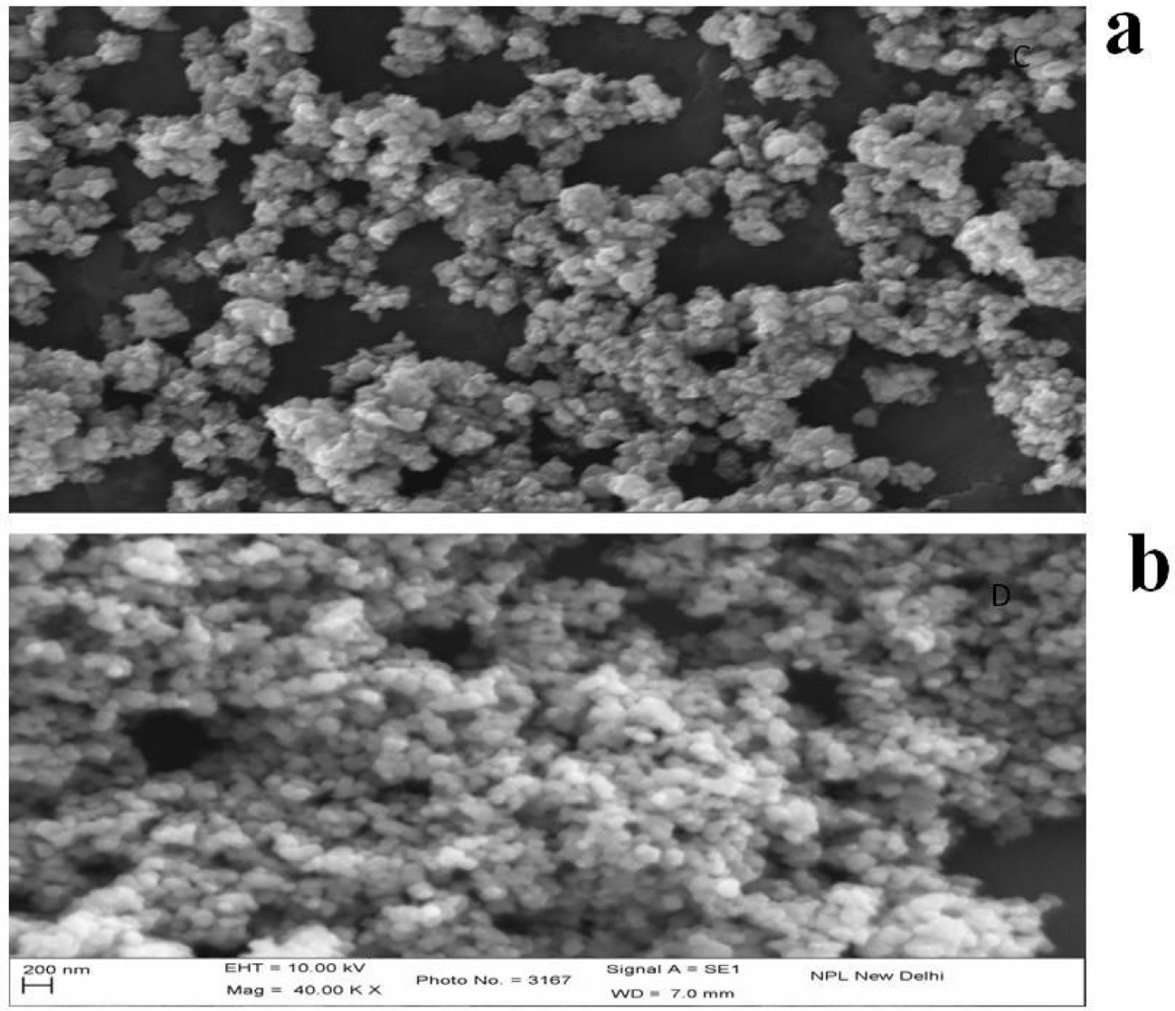
SEM images of synthesized (a) ZnO NPs and (b) TiO_2_ NPs.

The size and distribution of synthesized ZnO and TiO_2_ NPs were also confirmed by SEM shown in Fig. 5a-b. From the result it is evident that the morphology of ZnO NPs was irregular and TiO_2_ NPs was spherical in shape and polydisperse in nature, which is very similar to previous studies (Zahir et al., 2014; Rajakumar et al., 2012).

**Fig. 6:**
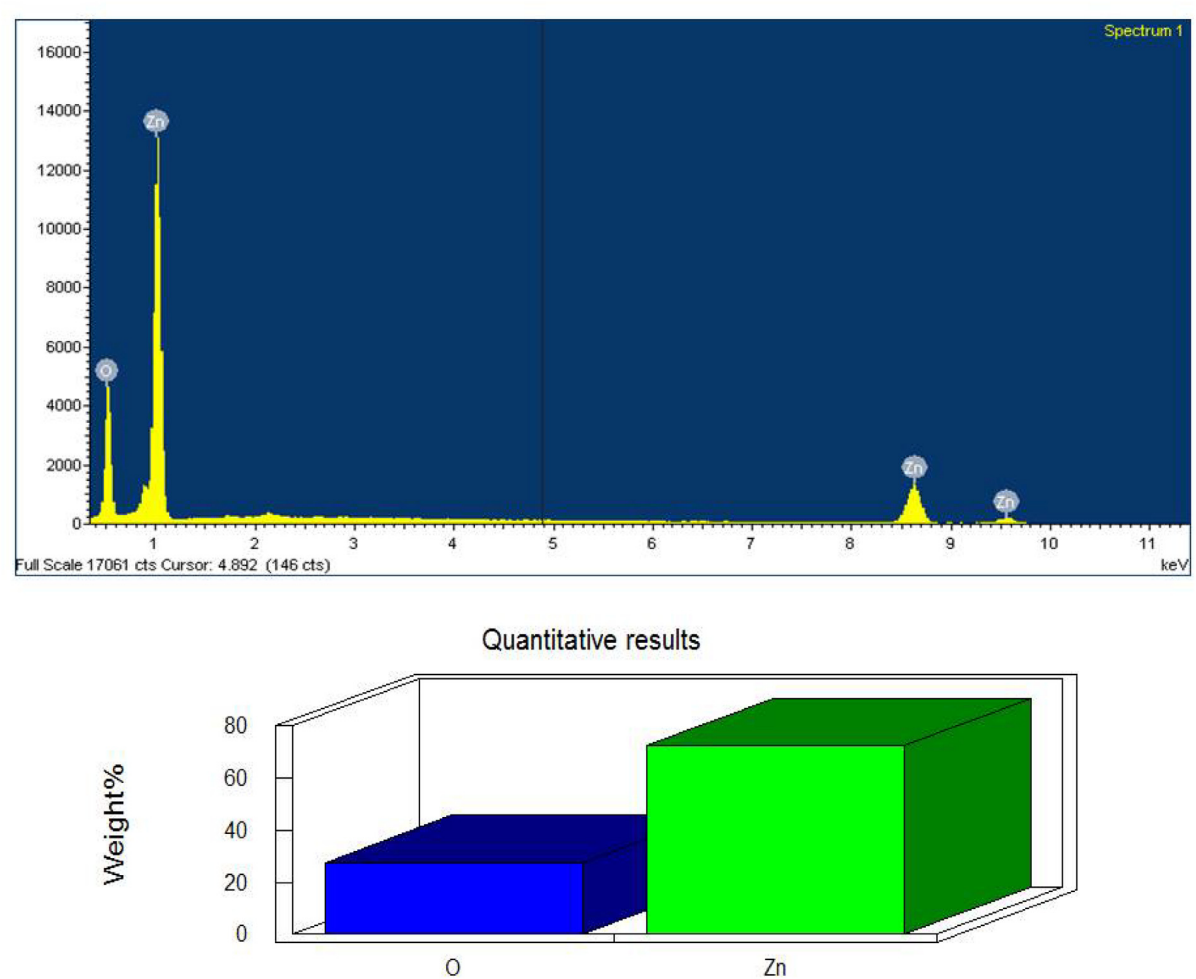
EDX images of synthesized ZnO NPs.

Fig. 6 shows the EDX analysis of ZnO NPs 72.57% zinc and 27.42% of oxygen which confirm the elemental composition of ZnO NPs. Narendhran and Sivaraj (2016) showed the EDX analysis of ZnO nanoparticles 37.22% of zinc and 62.78% of oxygen which confirms the elemental composition of ZnO nanoparticles. The strong signals from the zinc atoms in the nanoparticles recorded and other signals from C and O atoms were observed using EDX analysis in *Parthenium*-mediated ZnO nanoparticles (Rajiv et al., 2013). The EDX analysis display the optical absorption peaks of ZnO nanoparticles and these absorption peaks were due to the surface plasmon resonance of ZnO nanoparticles (Ankanna and Savithramma, 2011).

**Fig. 7:**
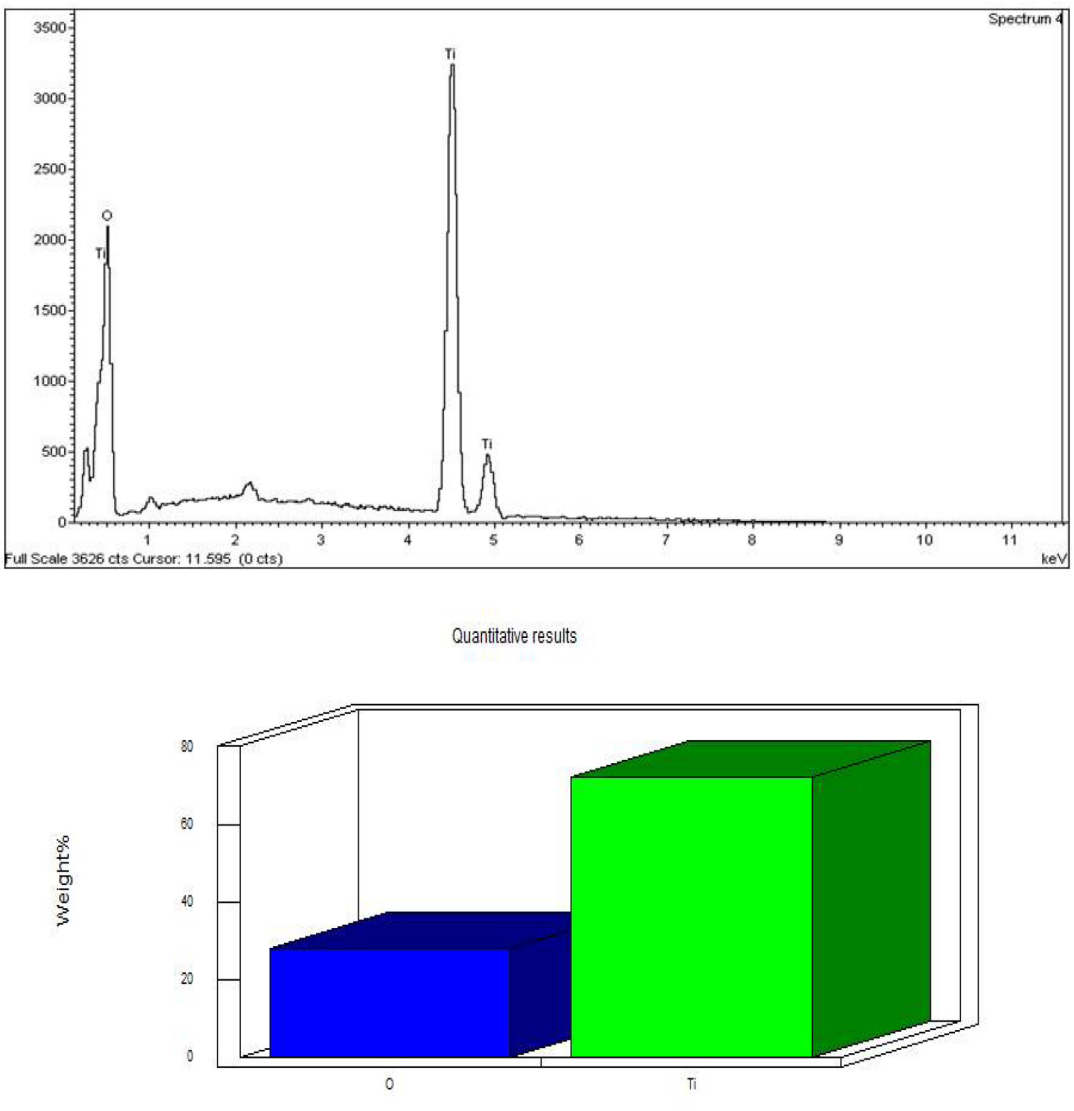
EDX images of synthesized TiO_2_ NPs.

Fig. 7 depicts the EDX analysis of TiO_2_ NPs 71.99% titanium and 28.01% of oxygen which confirm the elemental composition of TiO_2_ NPs. Santhoshkumar et al. (2014) showed the energy dispersive X-ray analysis study (EDX) which proves that the particles are crystalline in nature and indeed metallic TiO_2_ NPs. The similar results were reported in the previous studies by (Suman et al., 2015).

### 3.6 Larvicidal activity of ZnO and TiO_2_ NPs

Larvicidal activity of *F. religiosa* leaf extract, synthesized ZnO and TiO_2_ NPs were evaluated against the larvae (I-IV) of *An. stephensi* at different concentrations (25, 50, 100, 150 and 250 ppm).

The larvae of *An. stephensi* were found highly susceptible to the ZnO NPs. The fourth instar larvae have shown the 100% mortality after 24 h of exposure. Whereas, the first instar (LC_50_ 50 ppm), second instar (LC_50_ 75 ppm) and third instar (LC_50_ 5 ppm) larvae were observed with their probit equations and 95% confidential limit, R^2^, chi-square and *p* value after 24 h (Table 2). No mortality was observed in control group. The anti-parasitic activities have been assessed to determine the efficacies of synthesized zinc oxide nanoparticles (ZnO NPs) prepared by wet chemical method using zinc nitrate and sodium hydroxide as precursors and soluble starch as stabilizing agent against the larvae of cattle tick Rhipicephalus (Boophilus) microplus, Canestrini (Acari: Ixodidae); head louse *Pediculus humanus capitis*, De Geer (Phthiraptera: Pediculidae); larvae of malaria vector, *An. subpictus*, Grassi; and filariasis vector, *Cx. quinquefasciatus*, Say (Diptera: Culicidae) (Kirthi et al., 2011). The maximum efficacy was observed in zinc oxide against the *R*. *microplus*, *P. humanus capitis*, and the larvae of *An. subpictus*, Cx. *quinquefasciatus* with LC(50) values of 29.14, 11.80, 11.14, and 12.39 mg/L; r (2) = 0.805, 0.876, 0.894, and 0.904, respectively. The synthesized ZnO NPs showed the LC (50) and r (2) values against the *R*. *microplus* (13.41 mg/L; 0.982), *P. humanus capitis* (11.80 mg/L; 0.966), and the larvae of *An. subpictus* (3.19; 0.945 mg/L), against *Cx. quinquefasciatus* (4.87 mg/L; 0.970), respectively.

**Table 2:** Efficacy of *F. religiosa* aqueous leaf extract, Syntheiszed ZnO NPs and TiO_2_ NPs against the *An. stephensi* larvae with their probit equation, LC_50_ with 95% CL, χ^2^, p and R^2^ values.

	Instar	Concentrations (ppm)	% mortality	Probit equation	LC_50_ (±CL)	χ^2^	p	R^2^
Extract	I	25	0	y = 0.2203x + 0.6641	250 ± 1.22	1.95	0.744	0.903
		50	10					
		100	30					
		150	40					
		250	50					
	II	25	0	y = 0.2203x + 0.6641	250 ± 1.22	1.95	0.744	0.903
		50	10					
		100	30					
		150	40					
		250	50					
	III	25	30	y = 0.1016x + 28.32	200 ± 1.18	2.27	0.686	0.825
		50	30					
		100	40					
		150	50					
		250	50					
	IV	25	0	y = 0.3125x - 15.938	200 ± 1.14	1.54	0.820	0.919
		50	20					
		100	30					
		150	50					
		250	60					
ZnO NPs	I	25	40	y = 0.2297x + 35.586	50 ± 0.23	2.67	0.615	0.898
		50	50					
		100	60					
		150	70					
		250	80					
	II	25	40	y = 0.2297x + 35.586	75 ± 0.25	2.74	0.603	0.898
		50	50					
		100	50					
		150	80					
		250	90					
	III	25	50	y = 0.1859x + 42.617 2	5 ± 0.23	2.74	0.602	0.988
		50	50					
		100	60					
		150	70					
		250	90					
	IV	25	60	**	**	**	**	**
		50	90					
		100	100					
		150	100					
		250	100					
TiO_2_ NPs	I	25	60	y = 0.1188x + 62.344	15 ± 0.12	2.912	0.573	0.868
		50	70					
		100	80					
		150	80					
		250	90					
	II	25	40	y = 0.2141x + 37.383	50 ± 0.29	2.727	0.604	0.991
		50	50					
		100	60					
		150	70					
		250	90					
	III	25	50	y = 0.1234x + 49.805	25 ± 0.23	2.689	0.611	0.938
		50	60					
		100	60					
		150	70					
		250	80					
	IV	25	50	y = 0.1234x + 49.805	25 ± 0.34	2.710	0.608	0.938
		50	60					
		100	60					
		150	70					
		250	80					

** 100% mortality.

The TiO_2_ NPs were found effective against the larvae of *An. stephensi*. The mortality was recorded after 48 h of exposure. The first instar (LC_50_ 15 ppm), second instar (LC_50_ 50 ppm), third instar (LC_50_ 25 ppm) and fourth instar (LC_50_ 25 ppm) larvae were observed with their probit equations and 95% confidential limit, R^2^, chi-square and *p* value after 24 h (Table 2). The larvicidal activity of titanium dioxide nanoparticles (TiO_2_ NPs) synthesized from the root aqueous extract of *M. citrifolia* against the larvae of *An. stephensi*, *Ae. aegypti* and *Cx*. *quinquefasciatus* has been assessed (Suman et al., 2015). The biosynthesized TiO_2_ NPS showed maximum activity against the larvae of *An. stephensi*, *Ae. aegypti* and *Cx*. *quinquefasciatus* when compared to the aqueous extract of *M. citrifolia*. Similarly, the anti-parasitic activity of TiO_2_ NPs against the larvae of *R. microplus, H. anatolicum anatolicum* and *H. bispinosa*, fourth instar larvae of *An. subpictus*, and *Cx. quinquefasciatus* has been assessed by (Rajakumar et al., 2015). The maximum efficacy was observed in synthesized TiO_2_ NPs against the larvae of *R. microplus*, *H. anatolicum anatolicum*, *H. bispinosa*, *An*. *subpictus*, and *Cx. quinquefasciatus* with LC value of 28.56, 33.17, 23.81, 5.84, and 4.34 mg/L, respectively. Recently, the larvicidal and the pediculicidal activity of synthesized titanium dioxide nanoparticles (TiO_2_ NPs) using the leaf aqueous extract of *V. negundo* against the fourth instar larvae of the malaria vector, *An. subpictus* Grassi and filariasis vector, *Cx. quinquefasciatus* Say and the head louse, *P. humanus capitis* De Geer has been carried out by (Gandhi et al., 2016). The maximum activity has been observed in the synthesized TiO_2_ NPs against *An. subpictus*, *Cx. quinquefasciatus* and lice, (LC_50_ = 7.52, 7.23 and 24.32 mg/L; χ^2^ = 0.161, 2.678 and 4.495; r^2^ = 0.663, 0.742 and 0.924), respectively. The larvicidal activity of synthesized ZnO and TiO_2_ has been reported by other researchers (Table 3)

**Table 3:** Comparative larvicidal efficacy of synthesized ZnO and TiO_2_ nanoparticles against different mosquito species.

Plant species	Common name	Plant part used	Test NPs tested	Mosquito species	References
*Momordica charantia*	Bitter guard	Leaf	ZnO	*An. stephensi Cx. quinquefasciatus*	Gandhi et al., 2016
*Syzgium cumini*	Black plum	Seed	ZnO	*Ae. aegypti*	Roopan et al., 2018
*Scadoxus multiflorus*	Blood lily	Leaf	ZnO	*Ae. aegypti*	Abdullah Al-Dhabi and Valan Arasu, 2018
*Morinda citrifolia*	Noni	Root	TiO_2_	*An. stephensi Ae. aegypti, Cx. quinquefasciatus*	Suman et al., 2015
*Mangifera indica*	Mango	Leaf	TiO_2_	*An. stephensi Cx. quinquefasciatus*	Rajakumar et al., 2015
*Vitex negundo*	Chinese chaste tree	Leaf	TiO_2_	*An. stephensi Cx. quinquefasciatus*	Gandhi et al., 2016

The larvae of *An. stephensi* have also shown the mortality against the aqueous leaves extract of *F. religiosa* and mortality was recorded after 48 h. The first instar (LC_50_ 250 ppm), second instar (LC_50_ 250 ppm), third instar (LC_50_ 200 ppm) and fourth instar (LC_50_ 200 ppm) larvae were observed with their probit equations and 95% confidential limit, R^2^, chi-square and p value after 24 h (Table 1). The larvicidal efficacy of different extracts of *F. benghalensis* against *Cx. quinquefasciatus, Ae. aegypti* and *An. stephensi* has been investigated (Govindarajan, 2010). The lethal concentration (LC_50_) values of *F. benghalensis* against early second, third and fourth larvae of *Cx. quinquefasciatus, Ae. Aegypti* and *An. stephensi* were 41.43, 58.21 and 74.32 ppm, 56.54, 70.29 and 80.85 ppm and 60.44, 76.41 and 89.55 ppm respectively. Further, the larvicidal efficacy of different solvent leaf extracts of *F. benghalensis* against *Cx. tritaeniorhynchus* and *An. subpictus* has been determined (Govindarajan et al., 2011). The LC_50_ and LC_90_ values of *F. benghalensis* against early third instar of *Cx. tritaeniorhynchus* and *An. subpictus* were 100.88, 159.76 ppm and 56.66, 85.84 ppm, respectively. Thereafter, the Larvicidal activity of Indian medicinal plants, *C. berryi, C. pentandra, P. graveolens, T. peruviana, S.indicum, F. microcarpa, M. dubia, C. bonplandianus, F. religiosa* and *C. lacryma* has been tested against *Ae. aegypti* mosquito. They found the highest LC_50_ values of methanol extracts of *F. microcarpa* against *Ae. aegypti* larvae were 91.63 ppm followed by, LC_50_ values of *C. lacryma, P. graveolens, C. berryi*, and *M. dubia* extracts against *Ae*. aegypti larvae were 92.77, 95.65, 96.52 and 100.12 ppm, respectively.

### 3.7 Antibacterial activity

**Fig. 8:**
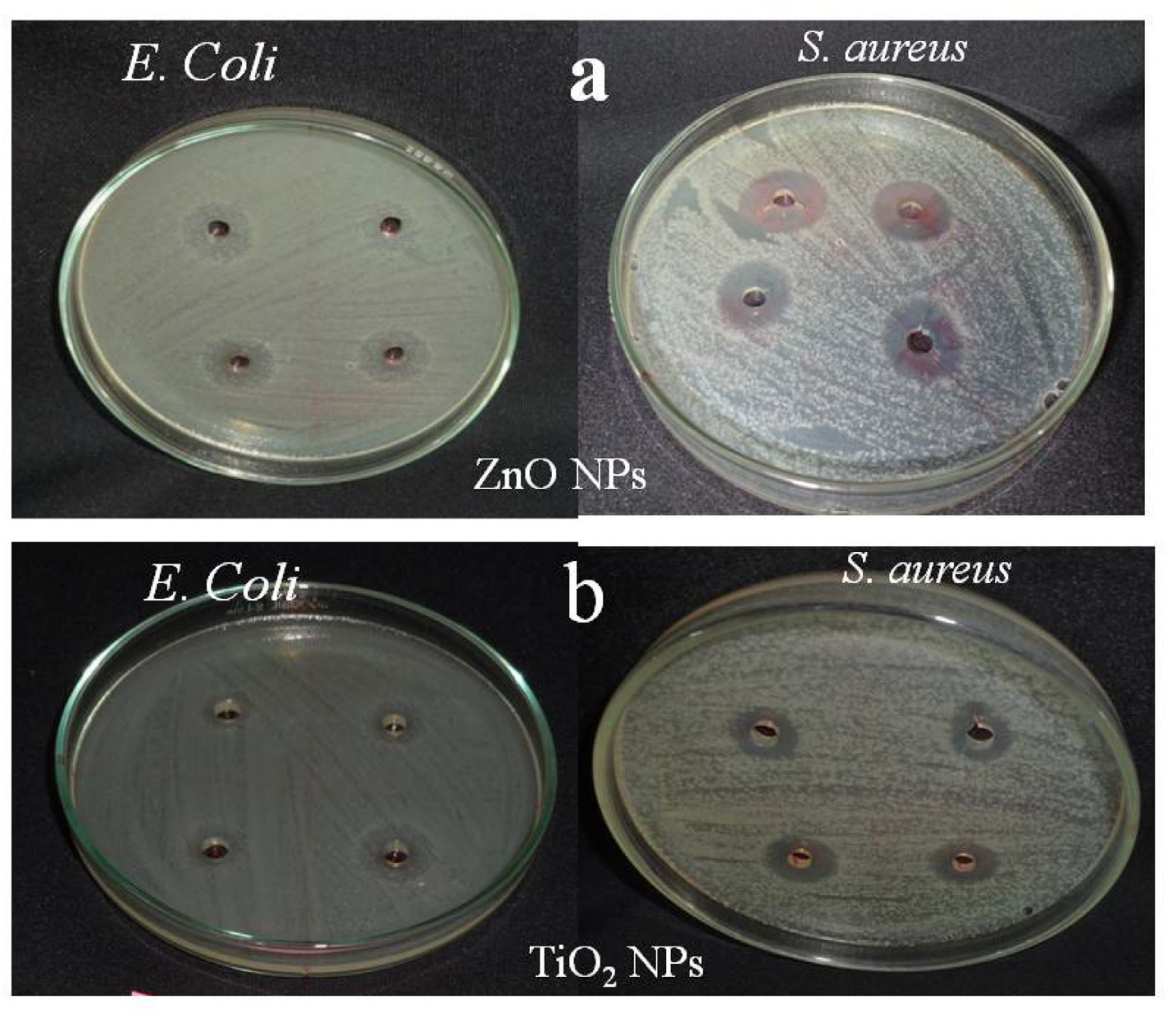
Antibacterial activity of synthesized (a) ZnO NPs and (b) TiO_2_ NPs against *E. coli* and *S. aureus*.

The antibacterial assay for biologically synthesized ZnO and TiO_2_ NPs against the pathogens is shown in Fig. 8. Well diffusion method was used to provide the evidence for and validate the antibacterial activity of ZnO and TiO_2_ NPs against *E. coli* (Gram negative) and *S. aureus*, (Gram positive) bacteria. The antibacterial activity of the ZnO and TiO_2_ NPs was indicated by the formation of the zone. The diameter of the inhibition zone was measured in millimetre. The maximum zone of inhibition was observed against ZnO NPs in *E. coli* (8, 10, 12 and 14 mm) and *S. aureus* (6, 8, 10 and 12) (Table 4 and Fig. 8). Several research confirming antimicrobial activity of ZnO NPs against the food related bacteria *B. subtilis, E. coli, P. fluorescens, S. typhimurium* and *S. aureus* has been reported (Russell and Hugo, 1994; Ip et al., 2006). ZnO NPs are also known to exhibit antimicrobial activities against L. *monocytogenes, S. enteritidis* and *E. coli* (Russell and Hugo, 1994). The formation of hydrogen peroxide from the surface of ZnO is considered to be mainly responsible for its antimicrobial property (Rai et al., 2009).

**Table 4:** Antibacterial activity of synthesized ZnO NPs and TiO_2_ NPs against *E. coli* and *S. aureus*.

	Species		Zone of inhibition/mm		
		50	150	250	500
ZnO NPs	*E. coli*	8 ± 0.612	10 ± 0.654	12 ± 0.712	14 ± 0.801
	*S. aureus*	6 ± 0.563	8 ± 0.612	10 ± 0.654	12 ± 0.712
TiO_2_ NPs	*E. coli*	7 ± 0.552	9 ± 0.642	10 ± 0.654	13 ± 0.752
	*S. aureus*	5 ± 0.456	6 ± 0.563	8 ± 0.612	10 ± 0.654

While, TiO_2_ NPs has shown the zone of inhibition in *E. coli* (7, 9, 10, and 13) and *S. aureus* (5, 6, 8 and 10) (Table 4 and Fig. 8). The higher zone of inhibition occurred at 500 ppm concentration of synthesized ZnO and TiO_2_ NPs. The antibacterial activity of TiO_2_ by pure plant extracts of *B. variegata* and *T. cordifolia* has been studied (Maurya et al., 2012). Plant extract/TiO_2_ nanocomposites have shown various level of antibacterial activity on different test microorganisms. The highest antibacterial potentiality expressed in terms of zone of inhibition (ZOI) in mm was exhibited by the aqueous extract of *B. variegata* /TiO_2_ (45 mm against *E. faecalis* and 30 mm against *E. coli*) and benzene extract of *T. cordifolia* /TiO_2_ (26 mm) nanocomposites. Similar results were reported (Kumar et al., 2014) using the biosynthesized and chemically synthesized titania nanoparticles and other researchers also (Table 5).

**Table 5:** Comparative antibacterial activity of synthesized nanoparticles ZnO and TiO_2_ nanoparticles against different microorganisms.

Plant species	Plant part used	NPs tested	Species	References
*Catharanthus roseus*	Leaf	ZnO	*B. thuringiensis, E. coli, S. aureus, P. aeruginosa*	Bhumi and Savithramma, 2014
*Brassica oleraceae*	Leaf	ZnO	*E. coli, V. cholera, C. Botulinum, S. aureus, B. subtilis*	Raj et al., 2015
*Trifolium pratense*	Flower	ZnO	*S. aureus, P. aeruginosa, E. coli*	Dobrucka and Dugaszewska, 2016
*Bauhinia tomentosa*	Leaf	ZnO	*B. thuringiensis, E. coli, S. aureus, P. aeruginosa*	Sharmila et al., 2018
*Pandanus odorifer*	Leaf	ZnO	*B. subtilis, E. coli*	Hussain et al., 2019
*Aloe vera*	Leaf	ZnO	*E. coli*	Izwanie Rasli et al., 2020
*Psidium guajava*	Leaf	TiO_2_	*E. coli, S. aureus*	Santhoshkumar et al., 2014
*Cynodon dactylon*	Leaf	TiO_2_	*E. coli*	Hariharan et al., 2017
*Glycosmis*	Leaf	TiO_2_	*S. saprophyticus, B. subtilis, E. coli, cochinchinensis P. aeruginosa*	Rosi and Kalyanasundaram, 2018
*Cassia fistula*	Leaf	TiO_2_	*E. coli, S. aureus*	Swathi et al., 2019

## 4 Conclusion

The present study, synthesis of ZnO NPs and TiO_2_ NPs from the *F. religiosa* is a green, rapid, cost-effective, non-toxic and eco-friendly approach for mosquito control as well as antimicrobial agent also.

## Declaration of Competing Interest

Authors declare that there is no conflict of interest.
